# Applying Microsatellite Multiplex PCR Analysis (MMPA) for Determining Allele Copy-Number Status and Percentage of Normal Cells within Tumors

**DOI:** 10.1371/journal.pone.0042682

**Published:** 2012-08-15

**Authors:** Carles Garcia-Linares, Jaume Mercadé, Bernat Gel, Josep Biayna, Ernest Terribas, Conxi Lázaro, Eduard Serra

**Affiliations:** 1 Institute of Predictive and Personalized Medicine of Cancer (IMPPC), Badalona, Barcelona, Spain; 2 Programa de Diagnòstic Molecular de Càncer Hereditari, Laboratori de Recerca Translacional, Institut Català d'Oncologia (ICO) – IDIBELL, L'Hospitalet de Llobregat, Barcelona, Spain; Deutsches Krebsforschungszentrum, Germany

## Abstract

The study of somatic genetic alterations in tumors contributes to the understanding and management of cancer. Genetic alterations, such us copy number or copy neutral changes, generate allelic imbalances (AIs) that can be determined using polymorphic markers. Here we report the development of a simple set of calculations for analyzing microsatellite multiplex PCR data from control-tumor pairs that allows us to obtain accurate information not only regarding the AI status of tumors, but also the percentage of tumor-infiltrating normal cells, the locus copy-number status and the mechanism involved in AI. We validated this new approach by re-analyzing a set of Neurofibromatosis type 1-associated dermal neurofibromas and comparing newly generated data with results obtained for the same tumors in a previous study using MLPA, Paralog Ratio Analysis and SNP-array techniques.

Microsatellite multiplex PCR analysis (MMPA) should be particularly useful for analyzing specific regions of the genome containing tumor suppressor genes and also for determining the percentage of infiltrating normal cells within tumors allowing them to be sorted before they are analyzed by more expensive techniques.

## Introduction

Cancer development and progression is partly driven by the acquisition of somatic genetic alterations [1]. Both DNA copy-number changes and copy-neutral events have traditionally been identified by detecting the presence of allelic imbalances (AIs). These are significant deviations from the 1∶1 allelic ratio of any heterozygous marker in a diploid cell, which are often reported as a loss of heterozygosity (LOH) when analyzing tumor-control tissue DNA pairs. Different techniques have been used to specifically detect copy-number changes in tumor DNA, such as semi-quantitative [2] or quantitative [3] PCR reactions, Multiplex Ligation-dependent Probe Amplification (MLPA) [4], Multiplex Amplicon Quantification (MAQ) [5] or array-CGH [6]. However, these techniques are not able to detect copy-neutral events, such as homologous recombination, which often reduce mutated tumor suppressor genes to homozygosity [7]. Fluorescent multiplex microsatellite PCR has been developed, semi-automatized and used in tumor AI analysis for a long time [8], and is still used now [9]. Determining the quotient between tumor-control tissue allelic ratios for a given heterozygous marker identifies AI. Guidelines on how to score and interpret AI analysis using microsatellite multiplex have been developed (see for instance [10]) as well as methodological modifications and statistical refinement (see for instance [11]). However, in order to differentiate whether an AI in a given locus is caused by a loss of DNA material or by a copy-neutral event, the use of an additional technique is required to determine the copy-number status of the locus. In addition, one of the problems of genetic analysis of tumors is the presence of high percentages of normal cells infiltrating the tumors. Since the development of microsatellite PCR for tumor AI analysis the presence of normal (non-AI) cells has been identified by the remaining signal of the lost allele, and grossly estimated by a simple allele ratio. This approximation is quite accurate in the case of an AI caused by a true loss of DNA material but it is not a good method in the case of copy-neutral events.

Today SNP-array analysis is the only single technique that can provide high resolution genome scale information on allele-specific copy-number, map and evaluate copy-neutral LOH, and accurately model the fractions of normal and tumor cells in tumor samples [12]. However, SNP-array analysis is not the preferred technique when analyzing just a few loci (e.g. to evaluate the status of specific tumor suppressor gene locus) or when performing a screening of many samples to make a tumor triage for further analysis, because of the high cost.

In this study we describe how, by applying simple calculations to an analysis of a microsatellite multiplex PCR (here referred to as MMPA), it is possible to obtain accurate information regarding not only the allelic imbalance status, but also the percentage of tumor-infiltrating normal cells and the locus copy-number status. This allows us to infer which mechanism is generating the AI: copy-loss, copy-neutral or amplification. To evaluate the MMPA performance we analyzed microsatellite data from Neurofibromatosis Type 1-associated dermal neurofibromas. These tumors are difficult to genetically analyze due to their high degree of cellular heterogeneity. 25% of neurofibromas exhibit AI in the *NF1* locus [13]. Of these, in 62% of the cases AI is produced by a copy-neutral event (homologous recombination in all of them) and in 38% it is produced by copy-number loss (deletion in all cases). We applied the MMPA calculations to a set of dermal neurofibromas already characterized in a previous study [13]. We compared the newly developed calculations in this report, with previous data obtained by applying MLPA, Paralog Ratio Analysis (PRA) and SNP-array in the same tumor samples.

## Results

### MMPA set up

We used a previously designed microsatellite multiplex PCR [13] to set up new reaction conditions and perform quality controls that allowed us to perform MMPA calculations proposed in this work (Table S1 and Figure S1, S2, S3, S4, S5, S6 and S8).

#### Proportionality between co-amplified amplicons

The MMPA calculations proposed here rely on comparing co-amplified microsatellites of control-tumor pairs. Proportionality between co-amplified amplicons must be maintained. In the MMPA set up shown here 22 cycles of PCR amplification ensured an adequate yield of each PCR amplicon while still maintaining an exponential reaction phase, guaranteeing proportionality among the different co-amplified amplicons for each independent multiplex PCR reaction (Figure S1).

#### Constant of proportionality (Kμ) among sample pairs and variation analysis

**Figure 1:**
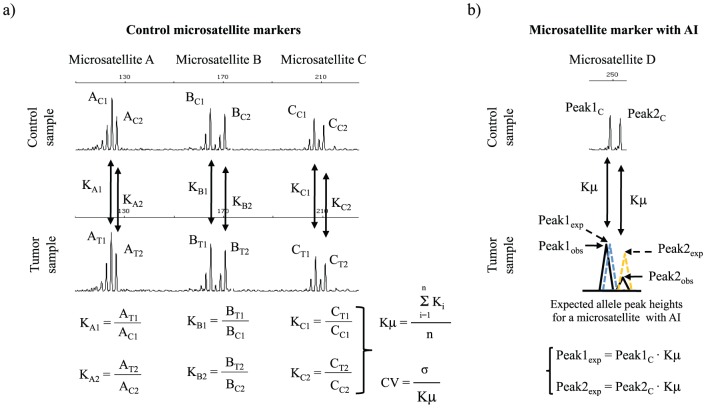
Constant of proportionality calculation and expected peak height estimation. a) Example illustrating how the average of the proportionality constant (Kμ) between two microsatellite multiplex PCR reactions that compare control and tumor paired samples, is calculated. Microsatellite electropherograms are shown. Control microsatellites are determined using Q^AI^ values (see text). Each microsatellite peak has a constant of proportionality (K). Kμ is the average of all individual Ks from control microsatellites. The coefficient of variation (CV) of Kμ is calculated as a quality control parameter (see text for details). b) Example of the calculation of expected peak heights (in the case 100% of the cells were non-AI) of a query microsatellite D with hypothetical allelic imbalance (AI) by using Kμ between control and tumor paired samples. Black line, observed peak heights (obs); dashed line, expected peak heights (exp).

Control-tumor independent multiplex PCR reactions can be compared and a constant of proportionality (K) can be calculated for each heterozygous microsatellite using peak height values (Figure 1a). Each K should be approximately the same for the different co-amplified amplicons within the MMPA reaction. The mean of the different microsatellite Ks (Kμ) reflects the constant of proportionality between the control-tumor compared reactions and can be calculated using either known pre-established control microsatellite markers within the MMPA reaction or by only taking into account those microsatellite markers that show no allelic imbalance (AI) in the tumor sample (see below). Variation of Kμ affects the accuracy and sensitivity of proposed MMPA calculations and the coefficient of variation (CV) of Kμ is used as a quality control (Figure 1a). Kμ variation should be set up and controlled for each MMPA before analyzing and comparing control-tumor pairs and we propose CV≤0.15 (Figures S2a and S2b).

#### Detection of allelic imbalances

AI analysis was based in the expression Q^AI^ [14] that represents the quotient between the tumor allelic ratio of a given heterozygous microsatellite marker within the MMPA reaction, and the allelic ratio for the same marker in the control sample [15]. A microsatellite marker was considered to show AI if, after calculating Q^AI^, there was a difference between ratios equal or greater than 0.2. This threshold was established after studying Q^AI^ variation caused by methodological errors (Figure S3).

#### Expected allele peak height values and comparison of observed vs. expected allele peak heights

**Figure 2:**
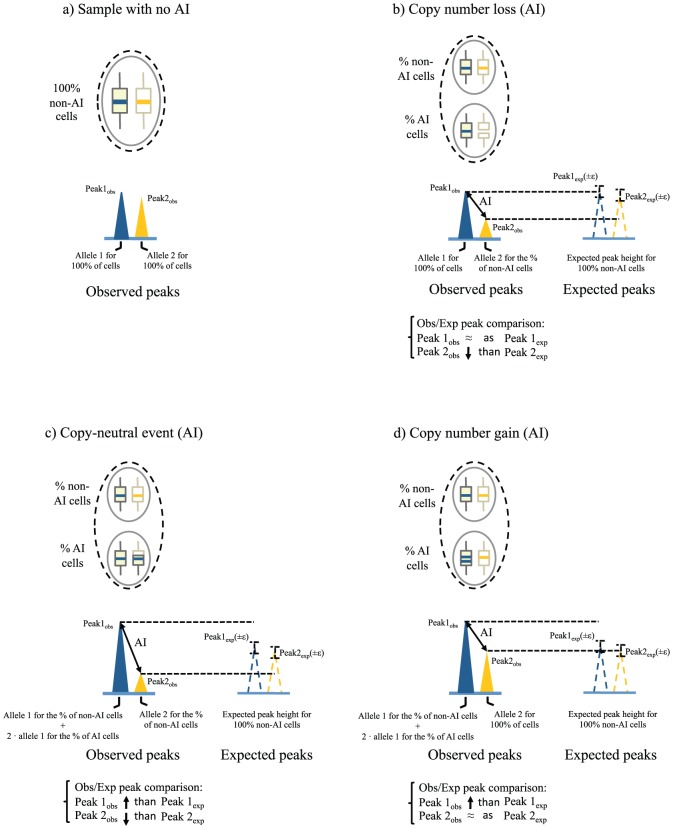
Peak_obs_ vs. peak_exp_ comparison for 1 heterozygous microsatellite in the analysis of AIs generated by different mutational mechanisms. Dashed line circles represent sample tissues (normal or tumor) and solid line circles represent types of genetically distinct cells within tissues. Blue and orange solid lines within chromosome-representing boxes indicate alleles of a heterozygous microsatellite marker. Solid color peaks represent allele peak heights obtained from microsatellite electropherograms after a theoretical MMPA. Dashed color peaks indicate expected peak heights in the case that 100% of the cells were non-AI, with the interval of peak height values indicated. Underneath allele peaks there is a short explanation of the source of peak height intensities regarding the number of amplified alleles according to the percentage of AI and non-AI cells. The Obs/Exp peak comparisons are also shown for the different mechanisms: a) Sample with no AI; b) Tumor sample with AI due to a copy-number loss; c) Tumor sample with AI due to a copy-neutral event; d) Tumor sample with AI due to a copy-number gain. Note that identical AI values for a given microsatellite in examples b and c will reflect distinct percentages of AI-cells within tumor samples.

MMPA calculations are based in the comparison between the observed peak height values of a heterozygous microsatellite marker (peak_obs_) with the hypothetical situation (expected peak heights, peak_exp_) in which there is no AI for that microsatellite marker. Peak_exp_ in the tumor is calculated by multiplying Kμ by the allele peak height value of the relative microsatellite in the control sample (peak_C_)(Figure 1b):
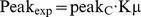
Allele peak heights of a microsatellite with AI will depend on the number of copies of that allele in AI-cells and the percentage of AI-cells within the tumor. Depending on the mechanism generating AI (Figure 2) peak_obs_ values will increase, decrease or will be equal to peak_exp_ values. By comparing peak_obs_ and peak_exp_ values of a given tumor microsatellite with AI, it can be ascertained which allele has lost a locus dosage (observed peak lower than expected), has an equal locus dosage (observed peak equal to the expected) or has a higher locus dosage (observed peak higher than expected) compared to the allelic status of the control sample pair of that microsatellite marker (Figure 2). To perform this comparison, an interval of peak_exp_ values is calculated by taking into account an empirically determined variation value (ε) that is applied to Kμ:
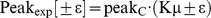
ε [ε = Kμ·Φ] depends and is intrinsic to each MMPA reaction set up and takes into account an upper limit threshold (Φ) established by analyzing individual deviations of K vs. Kμ for every microsatellite marker used in the MMPA reaction (see Figure S2b).

### Calculating the percentage of cells exhibiting allelic imbalance from AI-markers caused by copy-loss or copy-neutral events

**Figure 3:**
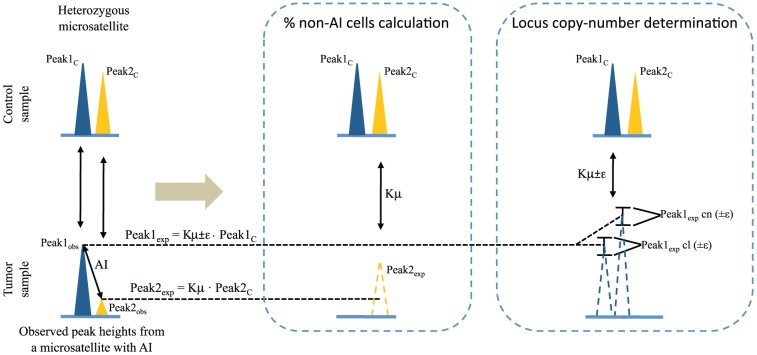
Calculating the percentage of non-AI cells and the locus copy-number of AI-cells from markers with allelic imbalance caused by copy-loss or copy-neutral events. Schematic view of the different use of both microsatellite alleles comparing observed vs. expected allele peak heights, concerning the calculation of the percentage of non-AI cells and the locus copy-number determination. Solid color peaks represent allele peak heights obtained from microsatellite electropherograms after a theoretical MMPA. Dashed color peaks indicate expected peak heights in the case 100% of the cells were non-AI. cl, copy-loss; cn, copy-neutral.

MMPA can be used to estimate the percentage of cells within tumors that carry an AI in an interrogated heterozygous marker locus. This is possible only in the cases where AI is produced by either copy-loss or copy-neutral events, and assuming a simple model in which tumors are mainly composed of two populations of cells: normal 2n cells and AI-cells. Once AI is identified, a comparison of peak_obs_ and peak_exp_ values is performed. In the case of AI due to copy-loss and copy-neutral events, one of the observed alleles will show a reduced peak height signal compared to the expected peak height (Peak 2_exp_ in Figures 2b and 2c and in Figure 3), and only this allele will be used to calculate the percentage of non-AI cells present in the tumor sample (Figure 3). The difference between the expected and observed peak heights is directly proportional to the percentage of cells bearing AI for this allele, since the observed peak height signal is only produced by the fraction of non-AI cells (heterozygous 2n cells) present in the tumor:
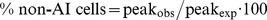
For simplicity, the calculation of non-AI cells present in a tumor will be performed using a single Kμ value, rather than an interval of values.

### Calculating locus copy-number in cells showing allelic imbalance from AI-markers caused by copy-loss or copy-neutral events

MMPA analysis can provide information on whether each allele of an AI-locus has one, two or more than two copies. Considering the same AI-marker, the allele not used for calculating the percentage of non-AI cells is used to determine both the number of copies of that allele and the mechanism generating AI (peak 1_obs_ in Figures 2b and 2c, and in Figure 3). Using the percentage of AI/non-AI cells previously calculated and taking into account gene dosages that will result from the different mechanisms generating AI, expected peak height ratios are calculated. ε will be used to generate interval values, considering two scenarios:
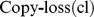


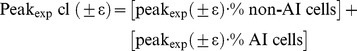


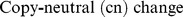


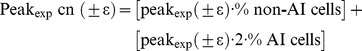
Peak_obs_ values will fit into one of the two intervals of peak_exp_, indicating the locus copy-number status and the mechanism generating AI. In the case peak_obs_ value does not fit any of the two expected intervals, the value should be considered out of range (OOR) and the corresponding microsatellite cannot be used for MMPA calculations. In the case that the two copies of a given locus were lost (e.g. nullosomy) or gained (e.g. tetrasomy) AI would not be detected. However, in these cases affected microsatellites would show K values substantially different from K values of other non-AI microsatellites.

A low degree of variation in Kμ for each MMPA reaction is critical for a correct assessment of both the percentage of AI/non-AI cells in tumors and allele copy-number (see Figure S4, S5 and S8).

### Analysis of other mechanisms generating AI in microsatellites

We have described so far the use of MMPA calculations for detecting copy-loss and copy-neutral events, but AI can also be due to copy-number gains of one allele or by the gain of a different number of copies of each allele (see Figure 2d as an example). In fact, equations described to obtain “peak_exp_ cl” or “peak_exp_ cn” could be adapted to calculate any gain in locus copy-number generating AI (see Fig. S6). When analyzing AIs produced by copy-number gains, information regarding the percentage of non-AI cells will not be produced. However, in the context of a specific tumor and under the assumption that all genetic alterations causing AI are present in the same tumor cell population, information on the percentage of normal cells present in that tumor could be obtained from other AI-markers if AI is caused by copy-loss or copy-neutral events. In the case that one or more extra copies of one allele is gained, peak_obs_ of that allele will be greater than the peak_exp_, while the remaining allele will be equal. In the case both alleles of an AI-marker gain extra copies but in different numbers, both peak_obs_ will be greater than the respective peak_exp_ (see Fig. S6 and S7).

### Applying MMPA analysis to determine the *NF1* locus status in neurofibromas

Our group previously characterized a large set of neurofibromas for the presence/absence of AI in the *NF1* region by applying microsatellite analysis [13]. In the same study, the copy-number status of the same region was determined by either MLPA, PRA or SNP-array analysis [13]. After developing the MMPA calculations presented here, we re-analyzed a set of 29 dermal neurofibromas with the newly established microsatellite multiplex PCR conditions and obtained data regarding the percentage of non-AI cells within these tumors and the *NF1* locus copy-number. We compared the new MMPA generated data with that previously obtained in [13] for these 29 neurofibromas (Tables 1 and 2).

**Table 1:** MMPA validation of the NF1 locus copy-number.

	MMPA calculations (present work)			Garcia-Linares et al. 2011		
Tumor samples	MMPA copy-number determination	Correct microsatellite determination/Total microsatellites with AI	Non determined microsatellites	(Copy-number assessment)		
				MLPA	PRA	SNP-array
P001-1N	Two copies	6/7	1 OOR	Two copies		
P009-1N	One copy	4/4		*		
P011-16N	Two copies	4/5	1 OOR	Two copies		Two copies
P022-19N	Two copies	3/5	2 OOR	Two copies		
P022-21N	Two copies	1/6	5 OOR	Two copies		
P023-14N	Two copies	6/8	2 OOR		Two copies	Two copies
P023-6N	One copy	3/4	1 RH	One copy	One copy	One copy
P023-97N	One copy	3/5	2 OOR	One copy	One copy	One copy
P030-2N	Two copies	4/5	1 OOR	Two copies		
P039-1N	One copy	4/4		One copy		One copy
P047-1N	Two copies	4/5	1 OOR	Two copies		
P054-1N	Two copies	6/6			Two copies	
P062-11N	One copy	2/2		One copy		
P079-1N	One copy	3/3		One copy		One copy
P081-1N	One copy	5/5		One copy		
P082-6N	One copy	5/5			One copy	One copy
P090-3N	One copy	3/3		One copy		One copy
P095-1N	One copy	2/3	1 OOR	One copy		One copy
P102-3N	One copy	4/5	1 OOR	One copy		
P102-4N	One copy	1/2	1 OOR	*		
P102-5N	Two copies	8/9	1 OOR	Two copies		
P102-18N	Two copies	9/9		Two copies	Two copies	Two copies
P103-5N	Two copies	5/5		Two copies	Two copies	Two copies
P103-21N	Two copies	4/6	2 OOR	Two copies		Two copies
P109-1N	Two copies	4/5	1 Del	Two copies		
P109-5N	Two copies	3/5	1 OOR, 1 Del	Two copies		
P109-6N	Two copies	4/4				Two copies
P109-7N	Two copies	3/4	1 OOR	Two copies		
P112-4N	Two copies	3/5	2 OOR	Two copies		

* see text for details.

Analysis of the copy number status of the *NF1* locus in 29 neurofibromas using newly developed MMPA calculations and comparison with previous data obtained for the same tumors using MLPA, PRA and SNP-array techniques (13).

**Table 2:** MMPA validation for the % of non-AI cells.

Tumor samples	MMPA (% non-AI cells)	SNP-array (% non-AI cells)	Difference between techniques (%)
P023-6N (Del)	40	42.5	2,5
P079-1N (Del)	66.1	66	0.1
P082-6N (Del)	57	65	8
P011-16N (HR)	67	74.9	7,9
P023-14N (HR)	45	59.5	14,5
P102-18N (HR)	43	47	4
P103-5N (HR)	44	50.6	6,6
P103-21N (HR)	56.1	55.7	0,4
P109-6N (HR)	46.2	53.6	7,4

Analysis of the percentage of non-AI cells present in 9 neurofibromas using newly developed MMPA calculations and comparison with data obtained applying GPHMM algorithm to previously generated SNP-array data for the same tumors (13).

In 26 out of 29 neurofibromas MMPA calculations correctly determined locus copy-number and AI-mechanism, considering all informative microsatellites (Table 1). In addition, AI-mechanism was correctly determined in all neurofibromas, considering that they had at least 3 informative AI-microsatellites (27/29), and taking into account a correct result when at least 60% of all informative microsatellites were indicating the correct mechanism (Table 1). Overall 144 microsatellites showing AI were identified in these 29 neurofibromas: AI-mechanism was correctly determined after MMPA in 116 (80.5%); 25 (17.4%) were out of range (OOR) meaning that the peak_obs_ value was not fitting within any of the expected intervals; and only 3 (2.1%) were incorrectly determined (Table 1). We included samples P009-1N and P102-4N (marked with an * in Table 1) in the analysis. These tumor samples contained high percentages of non-AI cells, making copy-number analysis impossible by MLPA, as shown in Garcia-Linares et al. (2011). However, they were considered to bear a deletion in the *NF1* locus, based in the similarity with other tumor samples bearing the same localized AI (only affecting the *NF1* gene and surrounding regions). MMPA calculations in these samples determined a deletion, supporting that this might be the actual mechanism generating AIs in these tumors. Copy-number ascertainment by MMPA calculations was possible due to the higher sensitivity of this technique when dealing with tumors containing high percentages of non-AI cells (Figure S5 and S8). Regarding the presence of normal cells within tumors, we calculated the percentage of AI/non-AI cells for 9 neurofibromas using previously generated SNP-array data [13] by applying the GPHMM algorithm [16]. We compared these data with the percentages obtained for the same tumors by applying MMPA calculations (Table 2). The results showed a good agreement between both techniques since, for all tumor samples but one, the calculated percentages of non-AI cells present in neurofibromas did not differ more than 8%.

In order to facilitate the calculations of the MMPA assay, we developed an automated analysis script that outputs the different parameters of an MMPA reaction together with the different calculations explained above (see Material and Methods; Script S1). This script is also freely downloadable at http://www.imppc.org/research-activities/genetic-variation-and-cancer/mmpa.html and can be easily customized to any MMPA design.

## Discussion

In this study we present a set of simple calculations (MMPA) applied to a microsatellite multiplex PCR to gain information on the percentage of normal cells present in a tumor, the copy-number status of specific alleles of heterozygous loci showing AI and the mechanisms that generate these AIs. Previously, to obtain the same type of tumor genetic information, the use of microsatellite PCR had to be complemented with additional techniques. A global view of this new approach is summarized in Figure S7.

All information that can be obtained by applying an MMPA is based on the use of a constant of proportionality (Kμ) between a multiplex PCR reaction of a tumor DNA and its matching control pair. Thus, one of the key factors when using MMPA calculations is to design and set up a robust multiplex reaction controlling all aspects that affect Kμ to minimize variation in its value and in that way minimize the errors in the estimation of expected peak heights (see supplementary information). DNA quality is also an important aspect for the correct performance of MMPA, as it could affect DNA amplification and increase Kμ dispersion. Bad quality DNA can make MMPA calculations impossible. In addition, it is important to precisely quantify and always analyze the same amount of DNA for all samples. K values from microsatellites with no AI considerably different from 1 could indicate genetic alterations causing copy-number gains or losses without generating allelic imbalances, as happens in cases where the two copies of a given locus were lost (e.g. nullosomy) or gained (e.g. tetrasomy).

Since Kμ is calculated from control microsatellites, it is desirable to have at least three informative control microsatellites in the MMPA. Previous knowledge about the degree of genetic instability and the recurrence of altered and non-altered genomic regions would facilitate the inclusion of pre-established control markers in the MMPA design. This should not be a problem when analyzing specific genomic regions in tumors with a low or intermediate degree of recurrent copy-number alterations. However, in tumors with a highly altered genome, it will not be possible to determine upfront which markers could be used as control microsatellites, and only after doing MMPA calculations could those not exhibiting AI (and Ks close to 1) be used as control markers.

In addition, during the MMPA analysis, special care should be paid to heterozygous dinucleotide microsatellites when both alleles only differ in one repeat (2 bp). In these cases, the slippage of the DNA polymerase during PCR amplification can hamper the correct analysis of peak height values of smaller alleles, thus affecting the calculations of copy-number and percentage of AI-cells in markers showing AI. In the MMPA setup presented here, we decided not to use heterozygous microsatellite markers with a difference in length of only 2 bp for the MMPA calculations. However in the case that these microsatellites were required, a correction on the peak height values of the smaller alleles could be applied [10].

The interpretation of the information produced by an MMPA is based on a simple model in which tumors are composed of two cell populations: normal 2n cells and cells containing allelic imbalances. A limitation of the MMPA in relation to the ascertainment of the percentage of normal cells present within a tumor is that this information can only be obtained from microsatellite markers showing AI due to copy-number loss or copy-neutral events. Then, this information can be used to analyze microsatellite markers showing AI due to copy-number gains present in the same tumor, but again, only under the assumption that all genetic alterations causing AI are present in the same population of cells. In tumors with a higher cellular and genetic heterogeneity not fitting with this simple model, AI could be detected but difficult to interpret in the context of copy-number and percentage of AI-cells.

The re-analysis of 29 neurofibromas showed a good agreement between the information generated by MMPA (copy-number status; percentage of AI/non-AI cells in the tumor) and the data generated in Garcia-Linares et al. (2011) using other standard techniques such as MLPA, PRA and SNP-array analysis. MMPA showed a high degree of sensitivity and accuracy. For the study of neurofibromas and the MMPA setup described here, most microsatellites (∼82%) showing AI were correctly determining the copy-number status and the AI-mechanism. ∼15% of microsatellites generated out of range values and thus, were finally not used in the analysis. The number of microsatellites indicating an incorrect AI-mechanism was very low (∼2%). Considering these data, using a criterion that allows the discrepancy of 1 AI-microsatellite, for neurofibromas with at least 3 informative AI-microsatellites (27/29) the MMPA analysis correctly determined the AI-mechanism in all of them.

MMPA calculations provide the same type of information that SNP-array analysis provides but at a much lower resolution. In contrast to this limitation, the cost is also much lower and after a brief PCR set up, it can be applied to any microsatellite multiplex PCR reaction already developed in any standard molecular biology lab. MMPA should be particularly useful for analyzing specific regions of the genome containing tumor suppressor genes causing cancer syndromes. Additionally it can be also used to scan different loci scattered along the genome in large series of control-tumor pairs to select a group of tumors for further and more refined analysis.

## Material and Methods

### Ethics Statement

All the human samples used in this study were provided by patients who had given their written informed consent. The ethical committee review board of the Bellvitge Hospital, Barcelona, Spain, approved the study.

### DNA extraction

DNA from blood was isolated either by the Wizard Genomic DNA purification kit (Promega) according to the manufacturer's instructions or by the “salting out” method as described elsewhere [17]. This DNA was used as control or reference DNA in the allelic imbalance analysis of tumors of the respective patients. Control DNA was also obtained in some cases from the skin of patients (or derived fibroblasts). DNA from skin or neurofibromas was extracted using the Gentra Puregene Kit (Qiagen) following manufacturer's instructions. Concentration, purity and quality of the DNA were first assessed using a nanodrop spectrophotometer and gel electrophoresis analysis. For an accurate quantification of DNA concentration, samples were further quantified using Quant-iT PicoGreen reagent (Invitrogen). All DNA samples were stored and preserved at 4°C and at −80°C. Samples used in this study: a) For the MMPA setting up samples are mentioned in Figures S1, S2a and S8; b) For MMPA validation 29 pairs of control-neurofibroma samples were used and are listed in Table 1.

### Microsatellite Multiplex PCR analysis (MMPA)

We have used a previously developed multiplex PCR reaction with the simultaneous amplification of 16 microsatellites ([13] and Table S1). To set up new conditions for performing the proposed MMPA calculations, we modified original PCR conditions according to the parameters and quality controls explained in the supplementary information. We used a Multiplex PCR Kit (Qiagen) with which we obtained good global microsatellite amplification, providing good peak heights and a robust band pattern in the electropherogram readings. We adjusted the concentrations of the different primer pairs to obtain similar product yields of each of the different amplicons of the multiplex PCR. The multiplex PCR reaction was carried out in a 25 µl reaction, containing 80 ng of DNA. An initial cycle of denaturation at 95°C for 15 min was followed by 22 cycles of: denaturation at 94°C, annealing at 56°C and extension at 72°C for 30 sec, 3 min and 1,5 min respectively. A final cycle at 60°C for 30 min was performed. 2 µl of PCR product was mixed with 7,8 µl of formamide and 0,2 µl of size standard Liz500. PCR fragments were separated by capillary electrophoresis on an ABI 3130*xl* Genetic Analyzer. Peak height values for each microsatellite allele were extracted using Peak Scanner software (Applied Biosystems). We used MMPA to analyze 29 control-neurofibroma pairs of samples.

### Semi-automated analysis of MMPA results

To analyze the data extracted by Peak Scanner software we have developed a tool that automates all the necessary MMPA calculations (Script S1). This automated analysis has been implemented using the Ruby programming language in a script named *mmpa.rb*. The script calculates and outputs the Kμ, comparing the reactions of control-tumor pairs, the average percentage of normal tissue contamination in the tumor, and the overall mechanism of AI for the test sample. For each informative microsatellite, calculated Ks, AI determination, observed peak height ratios, expected peak height ratios, % of non-AI cells and mechanisms generating the AIs are also calculated. *mmpa.rb* is released under the GNU GPL license. Source code and detailed instructions for its installation and use are available in Script S1 and can be also downloaded from http://www.imppc.org/research-activities/genetic-variation-and-cancer/mmpa.html.

### SNP-array data analysis

SNP-array data generated in a previous study [13] (Illumina Infinium SNP-array, Human660W-Quad beadchip ∼660000 SNPs) was used to determine the percentage of cells bearing AIs by applying a Global Parameter Hidden Markov Model (GPHMM) algorithm [16] that quantitatively models signal baseline shift due to aneuploidy and also the presence of normal diploid cells. Results were compared with MMPA calculations.

## Supporting Information

Figure S1 **All co-amplified microsatellites in the MMPA reaction need to be analyzed when still in exponential phase of amplification.** To determine the optimal number of cycles of the MMPA reaction, microsatellite amplification was monitored from 19 to 26 cycles. Peak height intensities for the two alleles of each microsatellite are plotted. This analysis was performed in triplicate for sample P114-15N (only one reaction is shown). We used 22 cycles for the MMPA technique exemplified in this work.
(EPS)

Figure S2 (**a**) We estimated a limit CV value (σ/Kμ) for the MMPA reaction exemplified here by analyzing Kμ variation in control microsatellites from four independent NF1 patients: 6 samples from patient P114, 4 samples from patient P027, 8 samples from patient P024 and 8 samples from patient P013. Each blue dot represents the CV of Kμ calculated for each pair of control samples (using one sample per individual as a reference). Only heterozygous microsatellites were used to calculate these ratios. As CV did not differ more than 0.1 in all but 2 cases, we considered CV ≤0.15 an optimal value for this MMPA reaction set up. (**b**) We performed an analysis to study the individual microsatellite behavior of the different co-amplified microsatellite markers in the MMPA reaction, and to obtain a cut off value on Kμ dispersion (Φ). Figure S2b shows the box-plots of 1-(K/Kμ), which measures the deviation of K with respect to Kμ for each co-amplified microsatellite marker allele. K/Kμ should be close to 1 in co-amplified microsatellites behaving similarly. Control samples used were the same as those in figure S2a, and values were generated for each pair of samples of the different individuals (using 1 sample of each individual as a control reference). For most microsatellite markers, K values didn't differ more than 12% from the Kμ value. We established Φ to be 0.12 in our MMPA reaction set up.
(EPS)

Figure S3 **We established a limit Q^AI^ value to determine the presence or absence of AI in tumor samples by analyzing the inherent variation of microsatellite allelic ratios in control sample pairs (here Q^AI^ = R_Cont1_/R_ Cont2_) using the expression 1-(Q^AI^).** Figure S3 shows box-plots of the 1-(Q^AI^) values of all ratios obtained from heterozygous microsatellites used in the exemplified MMPA reaction. For microsatellites with no AI, Q^AI^ should be close to 1 if there is no variation between allelic ratios. Control samples used were the same as those in figure S2a (using 1 sample as a control reference). For control samples with no AI, variation didn't exceeded 0.1. We established a limit Q^AI^ value of 0.2 to consider presence of AI.
(EPS)

Figure S4 **Methodological limitations of MMPA provide an inherent variation on Kμ values, as shown in Figure S2.** Figure S4 illustrates how variation on Kμ (by applying different CVs) affects the calculation of the % of 2n cells present in a tumor sample in a hypothetical reaction, for AI-microsatellites with one observed allele peak height lower than expected (copy-loss and copy-neutral events). In the presented hypothetical scenario the parameters used are: a Kμ = 1; a microsatellite with a control allele peak height of 1000 (fluorescence intensity); a tumor peak height representing a % of 1000 (fluorescence intensity) proportional to the % of non-AI cells present in the tumor. Solid line: % of 2n cells for CV = 0. Dashed lines: % of 2n cells for the different CVs applied. This figure shows that it is important to set up MMPA reactions with a low degree of variation on Kμ, since the greater the CV on Kμ the less accurate is the calculation of the percentage of 2n cells in the tumor.
(EPS)

Figure S5 **To determine the locus copy number of AI-microsatellites, two different intervals of expected allele peak height values are calculated, one for a copy-number loss scenario and another considering a copy-neutral event (see text for details and** **Figure 3** **).** The observed allele peak height will fit into one of the two expected intervals. However, depending on the percentage of normal cells present in the tumor and the Φ used, the two different intervals can overlap, making it impossible to discern which is the mechanism generating AI. Figure S5 shows at which percentage of 2n cells these two intervals overlap for a hypothetical MMPA reaction. The parameters used were: Kμ = 1, Φ = 0.12, control peak height = 1000 (fluorescence intensity), tumor peak height = 1000 for copy-loss (one copy of the locus) and 2000 for copy-neutral (two copies of the locus). Blue and green lines define the intervals (Kμ±ε; ε = Kμ·Φ) of expected allele peak height values for copy-loss and copy-neutral mechanisms respectively, under different percentages of 2n cells. This figure shows that the lower the Φ of the MMPA reaction set up, the higher the sensitivity for differentiating between copy-loss and copy-neutral events. This is important when analyzing tumors with high percentages of infiltrating normal cells.
(EPS)

Figure S6 (**a**) Schematic view of AIs generated by either single allele locus amplification (copy-number gain), or by differential amplification of both alleles (multiple amplification). Solid color peaks represent allele peak heights obtained from microsatellite electropherograms after a theoretical MMPA. Dashed color peaks indicate expected peak heights (in the case 100% of the cells were non-AI). A microsatellite marker with an AI generated by copy-number gain will show one of the observed allele peak heights higher than expected and the other one similar to its respective expected peak height. A microsatellite marker with an AI generated by multiple differential amplifications will show both observed allele peak heights higher than their expected peak heights. In both scenarios there is no observed peak height lower than expected. (**b**) Determination of allele locus copy-number for the two scenarios described. Only allele peak heights higher than expected will be used (Peak 1_obs_ in copy-gain and Peak 1_obs_ or Peak 2_obs_ for multiple amplification). In these cases, observed peak height values will fall inside the interval of expected peak height values [Peak_exp_ cg (±ε)] obtained by using the actual number of copies of the locus, represented by the variable X. The percentage of AI/non-AI cells used for these calculations will need to be obtained from a different microsatellite marker showing AI generated by either copy-loss or copy-neutral events.
(EPS)

Figure S7 **MMPA Workflow.**
(EPS)

Figure S8 **We performed an assay to determine the limit on the percentage of 2n cells present within tumor samples at which MMPA calculations are still reliable for the analysis of control/tumor pairs.** The example illustrates the case of an AI caused by a copy-loss. To reproduce this situation two different DNA samples were mixed at different proportions. One DNA was from a normal individual and the other was from his son with Neurofibromatosis type 1 caused by a deletion of the *NF1* gene (maternal origin) and adjacent regions (already characterized and published elsewhere). For all microsatellites located within the deleted region the son was missing the maternal allele and only the remaining paternal allele was present. DNAs were mixed raging from proportions of 0% father's DNA-100% son's DNA, to exactly the opposite (100% father's DNA-0% son's DNA), in steps of 10% increasing proportions of father's DNA. Alleles from heterozygous markers, outside the deleted region and shared by both father and son, were used as controls since, independently of the DNA admixture, they were always present in a 100% proportion (Figure S8c). To reproduce the presence of normal cells within tumors, heterozygous paternal alleles not present in the son and mapping within the deleted region were quantified (see boxed alleles in Figure S8c). A control sample with 100% father's DNA was independently paired with each of the different DNA admixtures. Kμ and the different calculations were generated for each pair of samples. The real percentages of DNA admixtures were compared to the calculated percentages obtained by applying MMPA. Copy number analysis and the mechanism generating AI was also determined. a) Results (in triplicate) of the % of non-AI cells and the mechanism generating AI obtained by applying an MMPA to the different DNA admixtures. MMPA technique was able to calculate the percentage of non-AI cells present in the DNA admixtures up to 70–80% while still detecting the presence of a deletion (see also Figures S4 and S5). b) Regression analysis indicating a good correlation between the real percentages of non-AI cells present in the DNA admixtures and the percentages calculated by applying MMPA. Blue dots represent the percentage of non-AI cells calculated using MMPA of each replica vs. the real percentage of non-AI cells. c) Examples of the electropherograms obtained for control and AI microsatellites for the different DNA admixtures.
(EPS)

Table S1 **Combination of primers used in the Microsatellite Multiplex PCR Assay exemplified in this work.**
(EPS)

Script S1 **Semi-automated analysis of MMPA results.** In order to facilitate the calculations of the MMPA assay, we developed an automated analysis script that outputs the different parameters of an MMPA reaction together with the different calculations. Script S1 contains files required to perform the MMPA analysis and a short Script guide.
(RAR)
